# Over-Expression of Human Lipoprotein Lipase in Mouse Mammary Glands Leads to Reduction of Milk Triglyceride and Delayed Growth of Suckling Pups

**DOI:** 10.1371/journal.pone.0020895

**Published:** 2011-06-17

**Authors:** Yuanyuan Wang, Jia Tong, Shuping Li, Ran Zhang, Li Chen, Yuhui Wang, Min Zheng, Meili Wang, George Liu, Yunping Dai, Yaofeng Zhao, Ning Li

**Affiliations:** 1 State Key Laboratory for Agrobiotechnology, College of Biological Sciences, China Agricultural University, Beijing, China; 2 Health Science Center, Institute of Cardiovascular Sciences, Peking University, Beijing, China; 3 GenProtein Biotech Ltd., Beijing, China; University of South Florida College of Medicine, United States of America

## Abstract

**Background:**

The mammary gland is a conserved site of lipoprotein lipase expression across species and lipoprotein lipase attachment to the luminal surface of mammary gland vascular endothelial cells has been implicated in the direction of circulating triglycerides into milk synthesis during lactation.

**Principal Findings:**

Here we report generation of transgenic mice harboring a human lipoprotein lipase gene driven by a mammary gland-specific promoter. Lipoprotein lipase levels in transgenic milk was raised to 0.16 mg/ml, corresponding to an activity of 8772.95 mU/ml. High lipoprotein lipase activity led to a significant reduction of triglyceride concentration in milk, but other components were largely unchanged. Normal pups fed with transgenic milk showed inferior growth performances compared to those fed with normal milk.

**Conclusion:**

Our study suggests a possibility to reduce the triglyceride content of cow milk using transgenic technology.

## Introduction

Lipoprotein lipase (LPL) plays a pivotal role in the transportation and energy metabolism of plasma lipoprotein, because it catalyzes the hydrolysis of the triglycerides (TG) circulating in chylomicrons and very low density lipoproteins (VLDL) into glycerol and non-esterified fatty acids (NEFA) [1], [2]. Functional LPL is anchored to the luminal surface of the capillary endothelium, where it is synthesized by parenchymal cells of adipose tissue, muscle, heart and the lactating mammary gland (MG). Recent study shows that a glycol protein, glycosylphosphatidylinositol- anchored high-density lipoprotein binding protein1 (GPIHBP1), also participates in the transport of LPL into capillaries via avid LPL binding [3].

Production of milk lipids by maternal MG in the mouse is equivalent to its entire body weight (BW) during a single lactation cycle (20 days) [4]. TG constitute 98% of milk lipid content and the 4% fat found in human milk provide 40–50% of total ingested calories [5]. Milk lipids are a vital source of energy and play an important role in the growth and development of mouse pups. TG cannot cross the capillary endothelium of most tissues, which suggests that LPL is involved in the uptake of blood TG by capillaries of mammary tissue for milk fat production [6] and that LPL activity levels reflect its capacity to direct TG from the blood [7]. Milk LPL is considered to be a spillover from MG and LPL activity in milk might indicate LPL activity in the MG. LPL activity in human milk is 200 n-equiv of fatty acid/min per ml, whereas the LPL activity level is 20-fold higher in mouse milk and six-fold higher in bovine milk [8].

Transgenic murine models have been widely used to study the tissue-specific function of LPL. Generalized over-expression of human LPL (hLPL) improved postheparin plasma LPL activity and reduced plasma TG in mice [9] [10], [11]. Transgenic mice that over-expressed LPL in skeletal muscle showed reduced plasma TG levels. Most of these mice exhibited weight loss [12], [13], but some maintained normal growth [14], insulin resistance was also observed [15], [16]. There are no previous reports of LPL over-expression in MG, which is the major tissue in production of milk lipids during lactation. We therefore aimed to establish a transgenic mouse model expressing human LPL (hLPL) in the MG. This model might be used to investigate the function of LPL in the MG and to evaluate potential applications in obtaining a low TG content cow milk in the future.

## Results

### Generation and Characterization of Transgenic Mice

**Figure 1:**
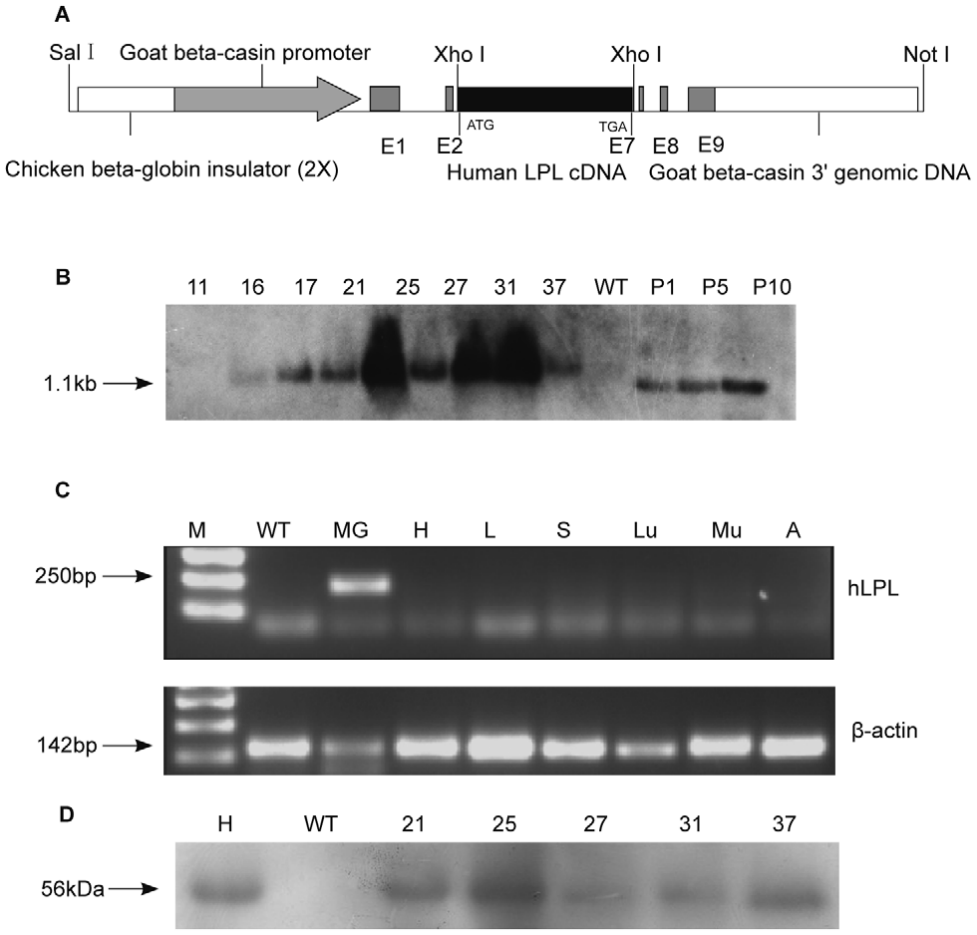
Generation and molecular characterization of transgenic mice. (A) hLPL cDNA (3.3 kb) was inserted into a backbone isolated from pBC1 using Not I/Sal I. The backbone included 2× chicken β-globin insulator, goat β-casein promoter, untranslated exons E1, parts of E2 and 7, E8, E9 and β-casein 3′ genomic DNA. hLPL cDNA was located between E2 and E7, flanked by two *Xho* I restriction sites. The translation initiation and termination sites are indicated as ATG and TGA. (B) Southern blot identification of transgenic mice. Digested genomic DNA of eight transgenic founders and WT mice hybridized with a 1.1 kb PCR labeled hLPL cDNA. An expected 1.1 kb band was detected in transgenic samples. WT, wild type mouse genomic DNA. P1, P5, P10 represent 1, 5 and 10 copies of hLPL cDNA plasmid, respectively. (C) RT-PCR analysis of hLPL expression in transgenic mice. All tissue RNAs used for RT-PCR were collected during the middle of the lactation period. Mouse β-actin was used as the RT-PCR internal control. M, 1 kb ladder; WT, wild type mouse mammary gland; MG, transgenic mouse mammary gland; H, heart; L, liver; S, spleen; Lu, lung; Mu, muscle; A, adipose tissue. (D) Western blot analysis of hLPL expression in milk of transgenic mice. The milk samples were collected during the middle of the lactation period, separated by SDS-PAGE and then transferred to nitrocellulose membranes. WT, milk from wild type mice (negative control); H, human milk (positive control); milk from transgenic founders numbered hLPL-21, -25, -27,-31 and -37.

Transgenic mice expressing milk hLPL were generated by inserting an hLPL cDNA into a pBC1 vector controlled by the MG-specific goat β-casein promoter (Figure 1A). Previous studies successfully used the pBC1 vector for high-level expression of the recombinant protein of interest [17], [18]. Eight transgenic founders (five females and three males) were identified initially by PCR and confirmed by Southern blot (Figure 1B). Further analysis showed that these transgenic founders habored different copy numbers of the transgene. Lines hLPL-11 (1), -16 (2), -17 (2) had only a few copies (one or two), whereas lines hLPL-21 (6), -25 (4), -27 (10), -31 (22), -37 (11) contained more copies (≥4). Copy numbers of the transgene varied from one to 22 copies per cell in founder lines.

We verified tissue-specific expression of hLPL controlled by the goat β-casein promoter by examining tissues taken from transgenic and wide type (WT) mice after 8 to 12 days of lactation, using RT-PCR. As expected, hLPL mRNA was detected in the MG of transgenic mice during the middle of the lactation period, but not in other tissues (Figure 1C). Western blot was further employed to detect recombinant hLPL in transgenic milk using an hLPL specific mouse monoclonal antibody (5D2). Milk samples were collected from five lines (hLPL-21, -25, -27, -31 and -37) of female transgenic founders during the middle lactation. Milk from all five transgenic founders contained the expected band of about 56 KDa, which is the molecular weight (MW) of native hLPL found in human milk (Figure 1D). hLPL was undetectable in milk of WT mice.

### LPL Concentration and Activity Assay

The ELISA kit we used did not specify whether it can distinguish recombinant human LPL from endogenous mouse LPL. We therefore compared the total LPL protein content in the milk between transgenic (n = 10) and WT mice (n = 6, Table 1). As such, LPL protein content in transgenic milk showed an approximately five- to six-fold increase, compared with control milk, during the different lactation stages. The highest level of LPL found in transgenic colostrum was 0.16 mg/ml. In general, LPL activity was high in the colostrum, but fell off during the lactation. There was 1.7-fold increase in LPL activity in transgenic milk at early lactation, 2-fold at middle lactation and 2.5-fold at late lactation, when compared with WT milk.

**Table 1:** LPL concentrations and activity in the milk of transgenic and WT mice during different lactation stages.

	LPL protein (**µ**g/ml)		LPL activity (mU / ml)	
	WT(n = 6)	hLPL(n = 10)	WT(n = 6)	hLPL(n = 10)
Early lactation	23.03±1.77	152.30±7.70***	4013.30±625.88	6923.30±1849.63*
Middle lactation	19.73±2.94	148.60±5.86***	2914.82±702.93	5872.96±1913.60*
Late lactation	21.65±1.60	127.91±22.24***	1993.46±270.77	4922.80±2018.19*

*P<0.05;

***P<0.001. All values represent means ± SD. n = number of animals.

### TG Dramatically Decreased in Transgenic Milk, but Not Total Protein or Lactose Content

**Figure 2:**
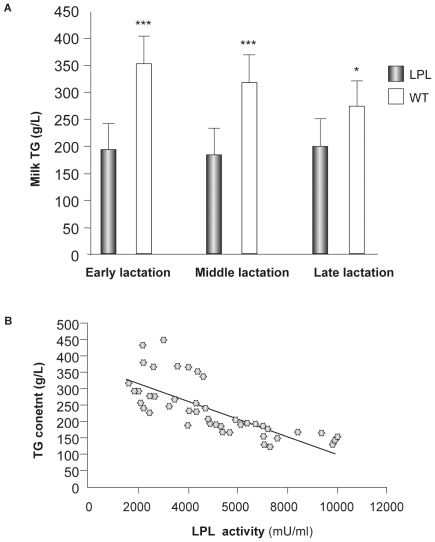
Change of TG concentration in transgenic milk. (A) TG content in milk of transgenic and WT mice during different lactation stages. Ten transgenic mice and six WT mice were tested. Bars represent mean (SD). (* P<0.05, *** P<0.001) (B) Correlation of TG concentration and LPL activity in milk of transgenic and WT mice during different lactation stages. (rho = 0.725 P<0.0001) [Data tested using Spearman's Rank test].

Milk samples were further analyzed for TG, total protein and lactose content. TG level in transgenic milk was significantly lower (nearly half) than WT milk throughout lactation (Figure 2A), which suggested a strong correlation between TG concentrations and LPL activity in the milk (Figure 2B). Analysis of milk composition revealed no change in lactose or total protein levels (Table 2).

**Table 2:** Composition of milk from transgenic and WT mice during different lactation stages.

(g / L)		LPL (n = 10)	WT (n = 6)
**Protein**	Early lactation	107.31±35.01	98.55±20.66
	Middle lactation	95.21±31.91	93.36±44.33
	Late lactation	101.12±18.80	116.93±7.87
**Triglyceride**	Early lactation	190.88±29.24***	352.84±65.99
	Middle lactation	182.59±39.70***	318.54±84.23
	Late lactation	199.37±62.23*	272.67±34.89
**Lactose**	Early lactation	15.40±4.60	16.36±4.50
	Middle lactation	11.62±4.25	9.70±4.34
	Late lactation	13.36±5.13	13.38±4.91

*P<0.05;

***P<0.001. All values represent means ± SD. n = number of animals.

### Free Fatty Acid Content in Milk and Serum

TG concentrations in the milk of transgenic mice decreased significantly compared with WT milk, so we determined whether reduced TG was hydrolyzed to FFA before being secreted into milk. Milk samples from all three stages of lactation were measured for FFA content. Quite unexpectedly, no significant difference of FFA content was observed between transgenic and WT milk (Figure S1). Levels of FFA in the serum were indistinguishable between transgenic and WT dams at middle lactation. We also analyzed the fatty acid composition of these milk samples, but found no different fatty acid carbon ratio between transgenic and WT milk throughout lactation (Figure S2).

### Growth Delay of Pups Fed by Transgenic Milk

In the early stage of this study, we noted that before weaning, pups reared by transgenic females seemed to grow more slowly independent of their genotypes (transgene or WT) compared with those reared by WT females. This indicated that the compositional change of transgenic milk might influence pup growth. We investigated this finding further using transgenic and WT dams, which were used to rear fostered newborns. This design was employed to exclude the influence of genetic background on growth. BW of pups selected at birth was approximately 2 g. Newborn pups from the same litter were divided into two halves, with one-half used for rearing by transgenic dams and the other by WT dams. In total six dams (three transgenic dams and three WT dams) were used in the experiment and each nursing dam was allowed to raise eight to ten pups.

**Figure 3:**
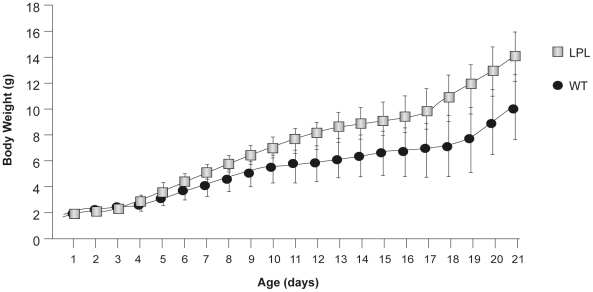
Growth curve of pups nursed from birth to day 21 by nursing mothers. Only litters of nursing dams which suckled newborns regularly were used in the statistical study. Body weight of each pup was weighed in the morning daily, from birth until weaning. Data were derived from a total of 30 WT pups belonging to three litters fed by three WT dams and 28 WT pups arising from three litters fed by three LPL dams. Error bars represent mean (SD).

There was a significant difference in BW after 5 days between pups reared by LPL (transgenic) and WT dams. Compared to pups nursed by WT dams (WT pups), pups nursed by LPL dams (LPL pups) showed retarded growth and their BW was always lower prior to weaning. At day 21 of lactation, the BW of LPL pups was approximately 10.18 g, which was 72.8% of WT pups BW (Figure 3). The difference between the heaviest and the lightest pups was 8 g. Maternal milk is the only nutrient source for suckling pups, so the great difference in pup BW must be attributable to differences in milk composition.

The most striking differences between transgenic and WT milk were LPL and TG concentrations, so either change might account for the observed differences in pup growth. To further address this issue, the gastric content of pups (3 days after birth) was tested to measure LPL activity. Dams and nursed pups were separated after six hours, and then reunited. Five minutes after pups began to suck milk, we collected milk curds in the stomach of pups to measure LPL activity. LPL activity for five LPL pups was 2.1, 5.6, 0, 1.0, 0 mU/mg, LPL activity for five WT pups was 1.0, 2.0, 0, 0, 0 mU/mg. This suggests that little LPL activity could be detected in the stomach of pups regardless of they were fed with transgenic or WT milk. Low pH conditions in the stomach probably led to LPL losing its activity. Our results strongly suggest that the retarded growth of pups fed by transgenic dams was due to the significant reduction of TG content.

Pup serum was collected to detect levels of TG, CHO and FFA at day 3, 10, and 20. As shown in Table 3, the TG level for LPL pups at day 3, day 10 and day 20, was 76.4%, 121% and 110%, respectively compared with WT pups. At day 10, the CHO level for LPL pups were 65.4% compared to WT pups. At day 3 and day 20, there were no significant differences in CHO levels between LPL pups and WT pups. We found no significant differences in FFA level between LPL pups and WT pups. Thus we found no pattern in the variation of fasting TG, CHO and FFA levels in LPL pups compared with the controls.

**Table 3:** Triglyceride, cholesterol and free fatty acid concentrations in the serum of pups aged 3 days, 10 days and 20 days which were nursed with transgenic and WT milk.

	WT			hLPL		
	3 days	10 days	20 days	3 days	10 days	20 days
**TG (mg/dl)**	365.00±109.64	245.4±42.41	286.5±46.13	279.10±25.34	297.90±21.97*	169.80±20.99***
**CHO (mg/dl)**	170.20±10.99	212.50±44.33	208.50±58.65	195.10±23.02	138.90±32.46*	158.40±12.99
**FFA (mEq/L)**	316.51±76.17	391.20±68.89	569.84±119.74	470.25±153.29	457.56±196.70	538.33±117.86

*P<0.05;

***P<0.001. All values represent means ± SD. n = number of animals.

### Nutrients in the Feces of Pups

**Figure 4:**
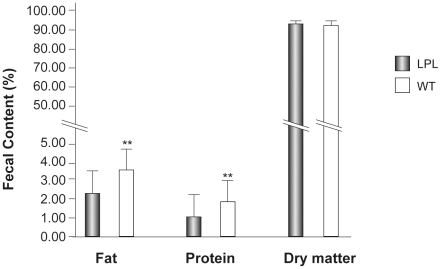
Fat, protein and dry matter measured in feces of suckling pups. Data shown are percentages of the total for the three constituents in feces. Bars represent mean (SD). (** P<0.01).

Feces were collected and analyzed for loss of nutrients from suckling pups. Figure 4 showed that crude protein levels were 1.02% to 1.10% for LPL pups and 1.60% to 1.92% for WT pups. Crude fat levels were 2.23% to 2.31% for LPL pups and 3.00% to 3.70% for WT pups. There was no significant difference in dry matter content between the two groups.

## Discussion

There are two putative sources of milk LPL. Firstly, LPL might be derived from tissues such as adipose tissue that secrete LPL from parenchymal cells before being transferred to milk [19]. Previous researches argued that if this was a viable route it might contribute little to milk LPL constitution [7]. Secondly, LPL derived from MG could be secreted into milk and also migrate through the basal membrane into the blood circulation. We found no hLPL in transgenic maternal serum by western blotting. However, the perennial argument about whether LPL is synthesized by the adipocytes or the epithelial cells of lactating MG was unresolved [20], [21]. We used an MG-specific promoter in this study to express protein exclusively in mammary epithelial cells and our results suggested that mammary epithelial cells can synthesize biologically active LPL. According to our result (Table 1), LPL mass in transgenic milk increased five- to six-fold compared to that in control milk, while LPL activity was only about doubled during various lactation stages. There is then a big drop in LPL specific activity in transgenic milk. This is most likely caused by the antibodies in the Elisa kit which have higher affinity towards exogenous human LPL than towards endogenous mouse LPL. Therefore, even though the real protein levels of exogenous human LPL might be similar to endogenous mouse LPL, the actual readings of human LPL protein levels would be much higher measured by this kit. However, the possibility that some inactive human LPL might be produced by transgenic mice can not be ruled out.

An interesting observation in this study was that the increased hLPL expression led to a dramatic reduction of TG content in the milk. The mechanism underlying this observation is not clear and might be explained by two possibilities. One is that hLPL produced by the transgenic mice competes with endogenous mouse LPL the binding sites at GPIHBP1 which is responsible for the transport of LPL into capillaries [6], leading to a limited capacity to transport LPL to the vascular endothelium where it directs the movement of fatty acid into the mammary cells. The other explanation would be that the over-expressed hLPL is able to hydrolyze the TG as it has been known that the LPL in goat milk can act on TG due to its presence in fat globule [22]. Hence, TG hydrolysis should occur around MG epithelial cells because this is the site of hLPL and TG synthesis in our transgenic mice. If this is the case, then we need to determine the destination of the TG hydrolysis products (FFA, etc). There are only two possible destinations; 1) secretion into milk and 2) entering maternal circulating blood. However, we detected no significant differences in the FFA content and FA composition of milk or maternal serum between transgenic mice and WT controls. FFA released by LPL hydrolysis appears to follow concentration gradients during transport [23] and is preferentially esterified for storage by tissues, or oxidized to provide an alternative energy source compared with plasma NEFAs [24]. It is probable that the FFA hydrolysis products of TG enter the maternal blood circulation and then are used for metabolism.

There is some controversy over whether milk LPL has a function in infant fat digestion. Previous researchers suggest that milk fat is not a good substrate for LPL, and LPL activity is also inhibited by bile salts in infants. Opponents of this view contend that LPL is critical for the assimilation of a high fat milk diet by suckling young [25]. In our study, LPL activity could not be detected in the stomach of suckling pups fed by either transgenic or WT milk. The loss of LPL activity was most likely due to low stomach pH, because the optimum pH for LPL activity is pH 8–9.

Fats are high energy sources and have a critical role in the growth and development of the neonates. Infants consume fats largely as TG, which are also integral constituents of neural and retinal tissues. Not surprisingly, low TG content in the milk caused significant reductions in the BW of suckling pup, because of a reduced energy supply. Significantly less fat and protein were found in the feces of LPL pups compared with WT pups, which might indicate that intestinal absorption of suckling pups was enhanced by the reduced TG content of milk.

In summary, we successfully generated transgenic mice that over-expressed hLPL, specifically in the MG. High LPL activity in the MG reduced milk TG during lactation. TG content was significantly reduced in transgenic milk, but the levels of other nutrients such as protein, lactose, and FA remained the same as the controls. Our study raises the possibility that low TG cow milk might be produced in future using the same method.

## Materials and Methods

### Animals

Kunming white mice (Beijing Laboratory Animal Center, CN) were used in this study. They were kept under conditions of regulated temperature (22 to 25°C), humidity (40 to 50%), and illumination cycles (12 h light, 12 h dark). They were provided with a standard rodent chow diet (1022 Rat/Mouse Maintenance Diet, HFK Bio-Technology Co. Ltd, CN) and water *ad libitum*. BW of pups was measured daily (from 09:00 to 10:30 h) throughout 21 days of lactation.

### Generation of Transgenic Mice

hLPL cDNA was amplified from a plasmid SC120026 (Origene, MD) by PCR, with *Xho* I restriction sites added at the 5′ end of both primers. The PCR product (3391 bp) was cloned into a T-vector and confirmed by sequencing. The cDNA fragment was inserted into a commercial pBC1 vector (Invitrogen, CA) at the *Xho* I site to generate the pBC-hLPL expression vector. pBC-hLPL was digested with *Not* I and *Sal* I to release a 19.1 kb linear DNA fragment, then was purified by agarose gel electrophoresis and electro-elution before microinjected into pronuclei of fertilized Kunming White mouse eggs.

### Genotyping of Transgenic Mice by PCR and Southern Blotting

Genomic DNA was isolated from tail tips of 3–4-week-old mice. The hLPL transgene was detected by PCR, using the upstream primer 5′-GTGGCTACCTGTCATTTCAAT-3′ and the downstream primer 5′-ACAGAGAGTCGATGAAGAG-3′, which yielded a 648 bp product. Eight transgenic mice (five females and three males) with hLPL cDNA in their genomes were identified from a sample of 42 mice.

Genomic DNA (15 g) was digested prior to southern blotting using *Eco*R I. A 1.1 kb hLPL cDNA fragment was amplified from the transgene vector (forward primer 5′-ATTCTGGATCTTTCGGACTGA-3′ and reverse primer 5′-ATTCCAAGCCTGATGATGTTA-3′) and used as a probe, which was labeled using a PCR digoxygenin probe synthesis kit (Roche, CH). Hybridization and detection methods were conducted in accordance with the manufacturer's digoxygenin hybridization instructions. Transgene copy numbers were determined by signal quantification using a phosphorimager (Storm820; Molecular Dynamics, Sunnyvale, CA).

### RT-PCR

Total RNA was isolated from the MG and other tissues (heart, liver, spleen, lung, adipose tissue, and muscle) with Trizol reagent (Tiangen, China). Two primers were used to detect transgene expression. The forward primer 5′-CCATTCAGCTTCTCCTTCA-3′ was complementary to exon 1 of goat β-casein on the backbone of pBC1 and the reverse primer 5′-AAATCTCTTCTTTGGTCGGC-3′ was complementary to the hLPL coding sequence. The two primers amplified a 250 bp PCR product. A 142 bp fragment amplified from mouse β-actin was used as an RT-PCR internal control. The forward primer was 5′-TTCTACAATGAGCTGCGTGTGG-3′ and the reverse primer was 5′-GGTGTTGAAGGTCTCAAACATGAT-3′.

### Milk Nutrient Assay and Western Blot

Maternal mice were anaesthetized with an intraperitoneal injection of pentobarbital (0.65 mg/10 g body wt.) and a 0.1 unit of oxytocin was delivered by intracardiac injection. Milk samples of transgenic mice and non-transgenic mice were collected during early (day 3–5), middle (day 8–12) and late (day 15–17) lactation. Milk samples were diluted three times with distilled water and separated by electrophoresis on 10% SDS-polyacrylamide gels before transfer to nitrocellulose membranes (Amersham Biosciences, US). Membranes were incubated overnight in blocking buffer (3% BSA in PBS-T) at 4°C, before immunodetection using a HRP-conjugated LPL (5D2) mouse monoclonal antibody (Santa Cruz, US) at a concentration of 1∶1000 as specified in the manufacturer's protocol.

Milk lactose concentration was determined using an enzymatic reagent kit (Catalog no. 10986119035) supplied by Boehringer (GER). Milk protein was measured as carcass protein [26].

### Assays for LPL Mass and Activity

Mouse milk LPL concentration was measured using a commercial ELISA kit (Catalog No. L040-90; GBD Ltd., US), according to the manufacturer's instructions. Milk LPL was assayed in duplicate, as previously described [27], using a [^3^H] triolein emulsion substrate. LPL activity of stomach curd milk was also tested using this method. One milliunit (mU) of lipase activity is equivalent to 1 nmol of free fatty acid (FFA) released per minute at 37°C.

### Lipid Analysis

Concentration of TG, cholesterol (CHO) and FFA in milk and serum was determined using commercial kits (Sigma, USA) (Catalog no. 99975406 WAKO Chemicals USA, Baltimore, Md.). Fasting (from 09:00 to 14:00 h) serum was collected for lipid measurement. Milk fatty acid composition was determined as previously described (Knapp 1979). All lipid samples contained a known volume of an internal standard. Results from gas chromatography were expressed as percentage composition.

### Fecal Analysis

Feces were collected from pups fed with WT and transgenic milk. All fecal samples were dried in a forced air oven (60°C) for 72 h and analyzed for fat, protein and dry matter, according to AOAC methods (1995).

## Supporting Information

Figure S1 **FFA concentrations in maternal milk and serum.** (A) FFA content in milk f transgenic and WT mice during different lactation stages. (B) FFA content in serum of transgenic and WT dam at middle lactation. Five transgenic and WT dam respectively were assayed after fasting. Bars represent means (SD).
(TIF)

Figure S2 **Fatty acid composition of milk.** Data shown are percentages of the total fatty acid content. The abscissa is on behalf of carbon ratio. Bars represent means (SD).
(TIF)
