# Supplementary figures and images for: Over-Expression of Human Lipoprotein Lipase in Mouse Mammary Glands Leads to Reduction of Milk Triglyceride and Delayed Growth of Suckling Pups
Source: PLoS One. 2011 Jun 17;6(6):e20895. doi: 10.1371/journal.pone.0020895 (PMC3117854; doi:10.1371/journal.pone.0020895)

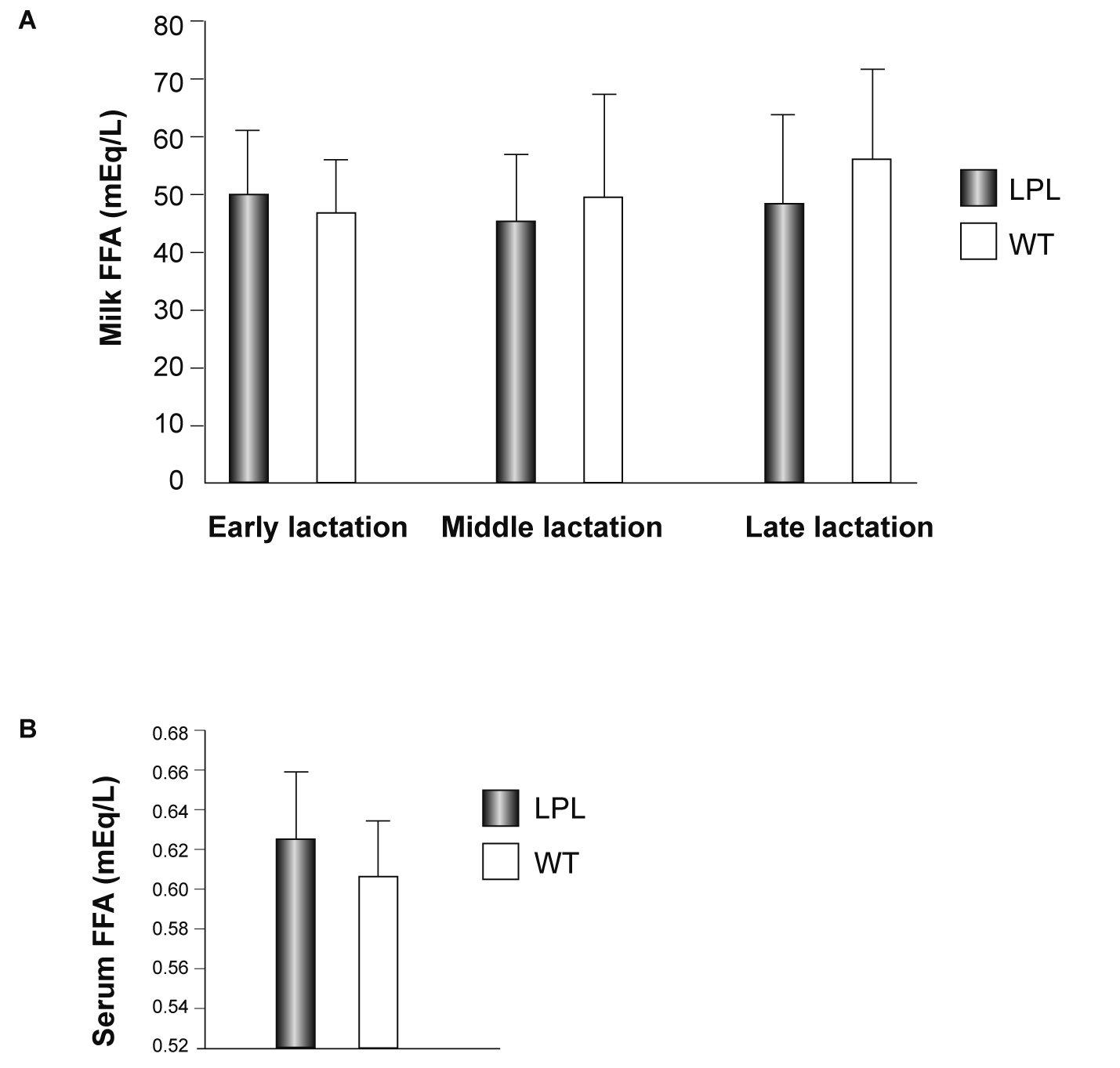

Supplement: Figure S1 — FFA concentrations in maternal milk and serum. (A) FFA content in milk f transgenic and WT mice during different lactation stages. (B) FFA content in serum of transgenic and WT dam at middle lactation. Five transgenic and WT dam respectively were assayed after fasting. Bars represent means (SD). (TIF) [file pone.0020895.s001.tif]

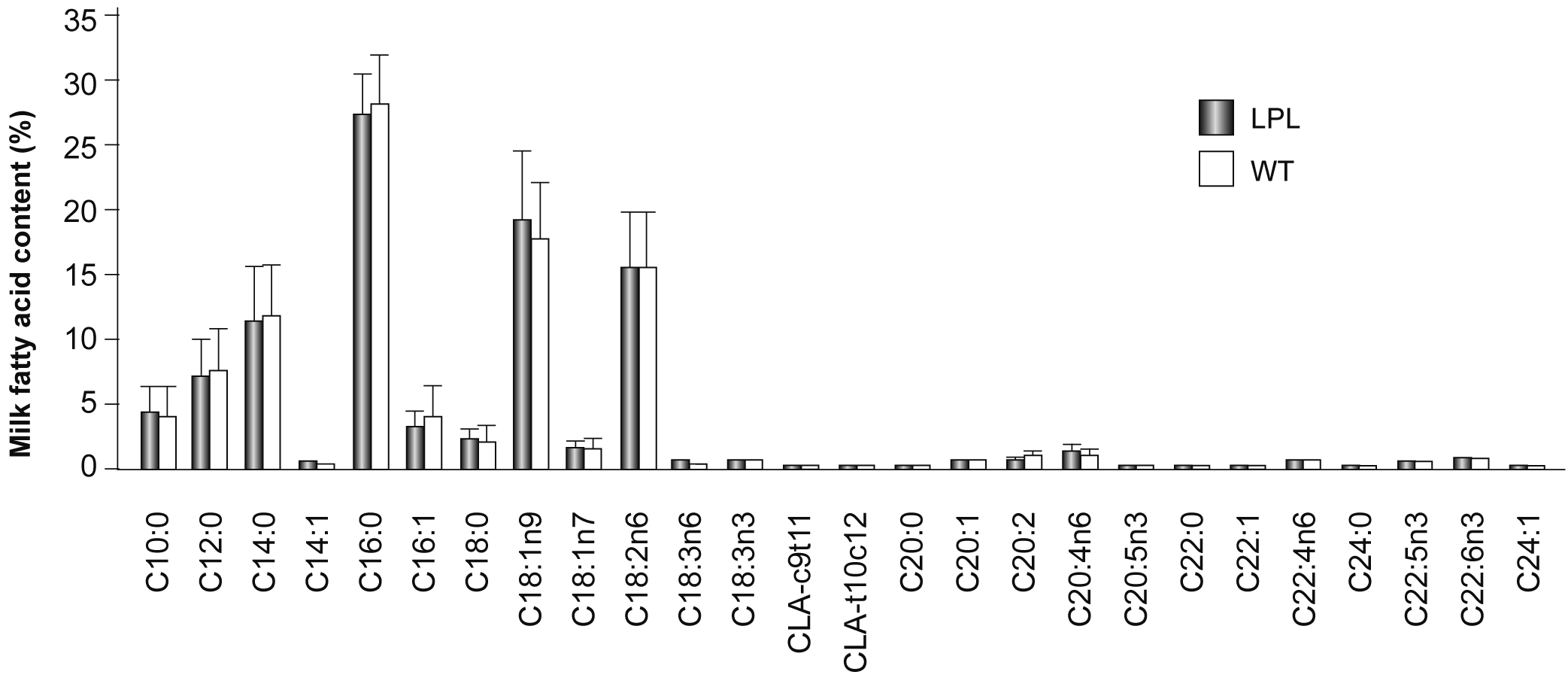

Supplement: Figure S2 — Fatty acid composition of milk. Data shown are percentages of the total fatty acid content. The abscissa is on behalf of carbon ratio. Bars represent means (SD). (TIF) [file pone.0020895.s002.tif]
